# Intranasal deferoxamine can improve memory in healthy C57 mice, suggesting a partially non‐disease‐specific pathway of functional neurologic improvement

**DOI:** 10.1002/brb3.1536

**Published:** 2020-01-20

**Authors:** Jared M. Fine, Jacob Kosyakovsky, Amanda M. Baillargeon, Julian V. Tokarev, Jacob M. Cooner, Aleta L. Svitak, Katherine A. Faltesek, William H. Frey, Leah R. Hanson

**Affiliations:** ^1^ Neuroscience Research at HealthPartners Institute Saint Paul MN USA

**Keywords:** deferoxamine, glycogen synthase kinase 3β, hypoxia‐inducible factor 1α, intranasal, memory enhancement, radial arm water maze

## Abstract

**Introduction:**

Intranasal deferoxamine (IN DFO) has been shown to decrease memory loss and have beneficial impacts across several models of neurologic disease and injury, including rodent models of Alzheimer's and Parkinson's disease.

**Methods:**

In order to assess the mechanism of DFO, determine its ability to improve memory from baseline in the absence of a diseased state, and assess targeting ability of intranasal delivery, we treated healthy mice with IN DFO (2.4 mg) or intraperitoneal (IP) DFO and compared behavioral and biochemical changes with saline‐treated controls. Mice were treated 5 days/week for 4 weeks and subjected to behavioral tests 30 min after dosing.

**Results:**

We found that IN DFO, but not IP DFO, significantly enhanced working memory in the radial arm water maze, suggesting that IN administration is more efficacious as a targeted delivery route to the brain. Moreover, the ability of DFO to improve memory from baseline in healthy mice suggests a non‐disease‐specific mechanism of memory improvement. IN DFO treatment was accompanied by decreased GSK‐3β activity and increased HIF‐1α activity.

**Conclusions:**

These pathways are suspected in DFO's ability to improve memory and perhaps represent a component of the common mechanism through which DFO enacts beneficial change in models of neurologic disease and injury.

## Graphical abstract

Intranasal deferoxamine (IN DFO) decreases memory loss and has beneficial impacts across several models of neurologic disease and injury. We show that IN DFO improves memory from baseline in normal healthy mice while modifying GSK‐3β and HIF‐1α, suggesting these pathways may be involved in a common mechanism through which DFO enacts beneficial change.

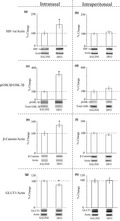

## 1 INTRODUCTION

Deferoxamine (DFO), a metal chelator with a high affinity for iron and other metal ions, has been shown to have beneficial effects in a variety of models of neurologic disease and injury. In vivo studies have demonstrated that DFO has protective effects in animal models of ischemic stroke (Freret et al., 2006; Hanson et al., 2009; Zhao & Rempe, 2011), intracerebral and subarachnoid hemorrhage (Hishikawa et al., 2008; Wan, Hua, Keep, Hoff, & Xi, 2006; Yu, Jia, & Chen, 2014), and traumatic brain injury (Zhang et al., 2013) and improves motor function and memory in Parkinson's disease (Fine et al., 2014; Kaur et al., 2003) and Alzheimer's disease (Fine et al., 2012, 2015; Hanson et al., 2012) models, respectively. Moreover, a clinical study found that DFO slows disease progression in patients with Alzheimer's disease (Crapper McLachlan et al., 1991). Studies also suggest that DFO may slow the process of retinal degeneration (Obolensky et al., 2011) and improve aspects of human cerebrovascular function, namely vasoreactivity and autoregulation, from baseline (Sorond et al., 2015).

The mechanisms and pathways through which DFO enacts beneficial change in these models have not been determined with certainty. While several disease‐specific changes were associated with DFO treatment in Alzheimer's and Parkinson's studies (Febbraro, Andersen, Sanchez‐Guajardo, Tentillier, & Romero‐Ramos, 2013; Fine et al., 2014; Kaur et al., 2003), several non‐disease‐specific biochemical changes, including chelation of redox‐active free iron, activation of hypoxia‐inducible factor‐1α (HIF‐1α), and its downstream targets, and inhibition of glycogen synthase kinase‐3β (GSK‐3β) via phosphorylation have been consistently correlated with administration of DFO (Fine et al., 2015; Sorond et al., 2015; Zhang et al., 2013; Zhao & Rempe, 2011). The commonality of these biochemical changes suggests that one or a combination of these pathways could play a major role underlying DFO's effects in all models.

Individually or in concert, DFO's chelation of free iron, its activation of the HIF‐1α pathway, and its inhibition of GSK‐3β likely contribute to its impact in neurologic disease states. A small fraction of total brain iron exists in a free ionic (or lightly chelated) state and is capable of causing neuronal injury through Fenton chemistry and the formation of damaging hydroxyl radicals, leading to oxidative stress (Zaman et al., 1999). Disruptions in iron homeostasis and iron overload are well characterized in the pathophysiology of neurodegenerative diseases such as Parkinson's and Alzheimer's diseases (Fine et al., 2014; Zecca, Youdim, Riederer, Connor, & Crichton, 2004). DFO's effects have also been postulated to be a direct by‐product of its activation of the HIF‐1α pathway, which is known to be involved in neuroprotection. HIF‐1α is a transcription factor that functions in the cellular response to hypoxia, resulting in the upregulation of neuroprotective downstream genes such as vascular endothelial growth factor and erythropoietin. HIF‐1α activity is highly regulated by prolyl hydroxylases and factor‐inhibiting HIF‐1, both of which require iron to function (Dongiovanni et al., 2008; Jacob et al., 2015; Schubert, Soucek, & Blouw, 2009). It has also been suggested that DFO's effects in neurodegenerative disease models may be related to its inhibition of glycogen synthase kinase (GSK‐3β), a proline‐directed serine/threonine kinase. GSK‐3β regulates neurogenesis, migration, axon growth and guidance, and synaptic plasticity, including long‐term potentiation (Salcedo‐Tello, Ortiz‐Matamoros, & Arias, 2011; Zhou & Snider, 2005). DFO's action has been consistently accompanied by significantly decreased GSK‐3β activity via phosphorylation at Ser9 (Fine et al., 2012, 2015). GSK‐3β has been strongly implicated in the processes of learning and memory (Hernandez, Borrell, Guaza, Avila, & Lucas, 2002; Hooper et al., 2007) as well as in neuroprotection (Leeds et al., 2014), and its inhibition could be a central contributor to DFO's impact in neurologic disease and injury.

It is unclear whether DFO acts through multiple disease‐specific mechanisms or exhibits its multiple effects through a common underlying non‐disease‐specific pathway. As an initial investigation into whether a diseased state is necessary for DFO's effects and mechanisms, we sought to determine whether DFO could enhance memory from baseline when administered to healthy mice—an effect that could only be a result of a non‐disease‐specific mechanism of memory improvement. Given that memory and learning enhancement from baseline has been well characterized with several other agents, including nicotine and nicotinic agonists (Levin, McClernon, & Rezvani, 2006), environmental enrichment (van Praag, Kempermann, & Gage, 2000), and insulin (Freiherr et al., 2013), we chose memory enhancement as the variable of interest. Another objective of this study was to determine the need for targeted brain delivery of DFO, comparing intranasal (IN), and intraperitoneal (IP) delivery routes at equivalent doses. IN delivery allows the targeting of therapeutics to the central nervous system along olfactory and trigeminal pathways while bypassing the blood‐brain barrier (Crowe, Greenlee, Kanthasamy, & Hsu, 2018). We administered DFO both IN and IP to normal C57 mice, performed radial arm water maze (RAWM) and Morris water maze (MWM) behavioral tests to assess their learning and memory, and examined brain tissues for changes in HIF‐1α and GSK‐3β to identify non‐disease‐specific effects. Ultimately, we found that IN DFO, but not IP DFO, enhanced working memory from baseline as measured in the RAWM and increased the ratio of both pGSK‐3β/GSK‐3β and HIF‐1α in the brains of healthy mice compared with saline controls.

## 2 METHODS

### 2.1 Experimental design

The study was conducted in two phases: (a) IN dosing with behavioral testing and (b) IP dosing with behavioral testing. For the behavioral studies, there were four treatment groups of mice: (a) IN DFO (10% solution); (b) IN saline; (c) IP DFO (10% solution); and (d) IP saline. There were 25 mice in each IN group and 14 in each IP group. Treatment groups were formed by partitioning mice into treatment groups of roughly equivalent average weight. After acclimation to handling, mice were given IN or IP dosing with coordinated behavioral testing with the MWM, RAWM, and open field test. Behavioral tests took place exactly 30 min after dosing for each mouse and started on the first day of behavior tests. The tests were administered Monday through Friday; testing with the RAWM also took place over the weekend. After behavioral testing, mice were dosed a final time with DFO or vehicle 30 min before euthanasia, and the tissues were collected for biochemical analyses.

### 2.2 Animal care and treatment

Seven‐week‐old male C57BL/6 mice were acquired from Harlan, Inc. Mice were group‐housed during a 12‐hr light/dark cycle and had continuous access to water and rodent chow. Dosing and behavioral testing were reserved for the day portion of the circadian cycle. All procedures were approved by the Animal Care and Use Committee of HealthPartners Institute at Regions Hospital under protocol #08‐024, and all experiments were performed in an AALAC‐accredited facility in accordance with all local and federal regulations.

### 2.3 Drug treatment and dosing

Deferoxamine mesylate salt was purchased from Sigma (D9533). It was administered to mice as a 10% solution (2.4 mg DFO/mouse or ~100 mg/kg) in 0.2× PBS (Sigma; P5493) at pH 6.0 for IN delivery. The vehicle was 0.2× PBS. For IP delivery, the same quantity of DFO (2.4 mg) was dissolved in 150 µl saline (0.9% NaCl) and administered. Awake mice were acclimated to handling and dosed IN as described in a detailed method paper by Hanson et al. (2012). Briefly, mice were first acclimated to handling and the IN delivery hold over the course of 3 weeks. They were then held in the supine position with a modified scruff and given a total of 24 µl with a micropipettor over 2.5 min. For IP dosing, mice were scruffed at the neck, held upside down, and given the drug or saline in the IP cavity. The 30‐min interval between dosing and behavior was chosen as it is a common time for compounds delivered intranasally to reach the brain (Thorne, Pronk, Padmanabhan, & Frey, 2004) and is functional for hypoxic preconditioning (Li et al., 2014).

### 2.4 Behavioral assessment

Behavioral tests were performed over 4 weeks. All behavioral tests were performed 30 min after drug delivery for each mouse. MWM was performed for 5 days in succession, while RAWM was performed 12 days in succession. Open field tests were conducted over the course of 1 day.

### 2.5 Radial arm water maze

Working memory was measured in a round, black plastic tub filled to a surface diameter of 108 cm with water at 21°C. Nontoxic white paint was added, and visual cues surrounded the tank. Six inserts were added to the tub to create 6 radially distributed, equal‐sized arms emanating from the center. Inserts were 41 × 15 cm long, and their walls extended 5.5 cm above the water surface. A clear platform measuring 6.4 cm^2^ was placed at the end of an arm 1 cm below the surface. The platform location was randomized (without repeating) each day. For 12 successive days, each mouse was tested in four successive acquisition trials in which it was placed at the end of a nonplatform arm and allowed to leave in search of the platform. An error was recorded when the mouse entered any of the five nonplatform arms or failed to make an arm choice within 20 s. After each error, the mouse was returned to its starting arm and released. Once the mouse located the platform or failed to do so in 60 s, it was allowed to stay on the platform for 20 s before the next trial. If a mouse had three or fewer errors and a 60‐s escape latency, it was assessed a penalty of eight errors to account for noncompliance. Errors and escape latency were recorded. The method was based on that originally developed in Arendash et al. (2001) and the same as that used in Fine et al. (2012) and Fine et al. (2015).

### 2.6 Morris water maze

Reference memory was assessed in the same tub and room as for RAWM but without the inserts, and a single platform was hidden 1 cm below the surface. Each day, mice were sequentially placed at the wall of the tub in 4 quadrants labeled 1 through 4, starting at the observer's left and distributed at each quarter of the tank, respectively. Each mouse had four trials/day. For each trial, mice were allowed to swim until they reached the platform or 60 s had elapsed, at which time they were then placed on the platform. All mice were given 20 s to rest before the next trial, and the platform remained at the same location for all trials. Data were videotaped and analyzed using the EthoVision tracking system (Noldus) for escape latency, path length, and velocity. After the hidden platform tests, a visual platform test was conducted to assess visual acuity. The platform was moved to a new location, raised just above the water level, and marked with a flag, and mice were given four trials during 1 day. Methods were the same as those used in Fine et al. (2012) and Fine et al. (2015).

### 2.7 Open field

Activity and exploration were measured in an open field, which was a white rectangle (85 cm [l] × 77 cm [w] × 28 cm [h]). Mice were placed in the center of the floor and allowed to explore for 5 min. Video was acquired with an overhead camera connected to a DVD recorder. The EthoVision tracking system (version 3.1; Noldus) was used to divide the area of the box floor into 16 boxes of equal size, and the total number of line crossings and velocity were recorded.

### 2.8 Euthanasia and tissue collection

All mice were anesthetized with pentobarbital, transcardially perfused with saline, and decapitated. Blood was processed with a serum separator tube (BD Microtainer), and serum was snap‐frozen in liquid nitrogen. The brain was removed from the skull and hemisected sagitally. The hippocampus was removed and snap‐frozen in liquid nitrogen. The tubes were stored at −70°C until analysis.

### 2.9 Protein extraction

Frozen brain tissues were homogenized in five volumes of ice‐cold RIPA buffer (50 mM Tris‐HCl, pH 7.4, 150 mM NaCl, 2 mM EDTA, 1% sodium deoxycholate, 1% NP‐40, 0.1% SDS) supplemented with protease inhibitor cocktail (Roche) and phosphatase inhibitor cocktail (Sigma). Homogenates were centrifuged at 20,000 *g* for 20 min at 4°C. Supernatant was stored at −70°C until analysis by Western blot.

### 2.10 Western blot analysis

Protein concentrations were determined using the bicinchoninic acid method. Equal amounts of total cellular proteins (50 μg for HIF‐1α and 25 μg for all other protein targets) were diluted in Laemmli buffer, separated by SDS‐PAGE, and transferred to polyvinylidene fluoride membranes. The membranes were blocked with 5% dry milk in Tris‐buffered saline/0.1% TWEEN (TBS‐T) overnight at 4°C on a shaking platform. Membranes were then incubated for 1 hr with 1 of the following antibodies in TBS‐T: phospho‐GSK‐3β (Ser9) rabbit antibody (Cell Signaling Technology, cat. #9336, RRID:AB_331405), GSK‐3β (27C10) rabbit monoclonal antibody (Cell Signaling Technology, cat. #9315, RRID:AB_490890), HIF‐1α rabbit polyclonal antibody (Novus Biologicals, cat. NB100‐479, RRID:AB_10000633), GLUT‐1 rabbit polyclonal antibody (Abcam, cat. ab652, RRID:AB_305540), and β‐catenin E‐5 (Santa Cruz Biotechnology, cat. sc7963, RRID:AB_626807). Rabbit anti‐actin polyclonal antibody was detected on all blots as a loading control (Novus Biologicals, cat. NB600‐503, RRID:AB_10077516). Membranes were rinsed in TBS‐T and then incubated in either anti‐rabbit (Cell Signaling Technology cat. #7074; RRID:AB_2099233) or anti‐mouse (Cell Signaling Technology, cat #7076, RRID:AB_330924) IgG conjugated to horseradish peroxidase in TBS‐T with 5% dry milk for 1 hr. Enhanced Chemiluminescence Plus Western blotting detection reagent (GE Healthcare) was used to visualize peroxidase enzymatic activity on X‐ray film. Exposed films were quantified using ImageJ software provided by the National Institutes of Health.

### 2.11 Statistical analyses

Statistical analyses for escape latency in the RAWM consisted of a Mann–Whitney *U* test due to violations of the assumption of normality (due to a ceiling effect of 60 s maximum to find the platform). *t* Tests were used for number of errors. Repeated‐measures ANOVA was performed to analyze MWM data. Both RAWM and MWM performance of IN and IP DFO treatment groups were independently compared with IN and IP saline control groups, respectively, because they were run at different times and therefore could not be directly compared to each other. Other behavioral testing and all Western blots were analyzed via *t* tests between treatment groups. Again, IN and IP groups were only compared with their respective controls.

## 3 RESULTS

### 3.1 Radial arm water maze

**Figure 1:**
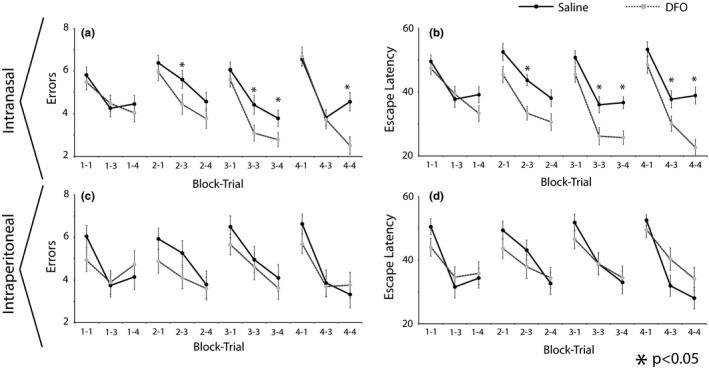
Radial arm water maze data for mice treated with DFO delivered IN (a, b) or IP (c, d) daily for 1 month. Behavioral tests were done 30 min after drug delivery each day. Data includes errors (a, c) and escape latency (b, d). Each block is an average of 3 days, and data are shown for trials 1, 3, and 4. IN DFO‐treated mice had a significantly shorter escape latency and fewer errors than saline‐treated mice in several trials (*p < .05), indicating that IN DFO treatment enhanced learning. There were no significant differences between IP DFO‐ and IP saline‐treated mice for any trials. Treatment groups for IN and IP delivery of DFO are only compared with their own controls, respectively, as IN and IP trials were not done concurrently

*t* Tests performed for each trial in each block showed that mice treated with IN DFO had significantly fewer errors when compared to IN controls in several cases during trials 3 and 4 (*p* < .05) (Figure 1a). These differences were not evident when comparing IP DFO with IP Saline treatment at an equivalent dose (Figure 1c). Similarly, Mann–Whitney *U* tests performed for each trial in each block showed that mice treated with IN DFO had significantly shorter escape latencies than control mice during trials 3 and 4 (*p* < .05) (Figure 1b). Again, these differences were not present in the case of mice treated with IP DFO (Figure 1d).

### 3.2 Morris water maze

There were no statistically significant differences between the IN or IP DFO treatment groups and their respective controls in escape latency, path length, or velocity (data not shown). This applies to both hidden and visual platform tests.

### 3.3 Open field

There were no statistically significant differences between any groups in either line crossings or velocity (data not shown). Mice treated with IN DFO had, on average, 82.1 ± 3.4 line crossings and average velocity of 7.3 ± 0.2 cm/s, while IN saline mice had, on average, 83.7 ± 4.6 line crossings and average velocity of 7.4 ± 0.3 cm/s. Averages for IP DFO mice were 94.7 ± 6.8 line crossings and a velocity of 8.3 ± 0.4 cm/s, while IN saline mice had, on average, 82.3 ± 5.0 line crossings and average velocity of 7.5 ± 0.4 cm/s.

### 3.4 Western blot

**Figure 2:**
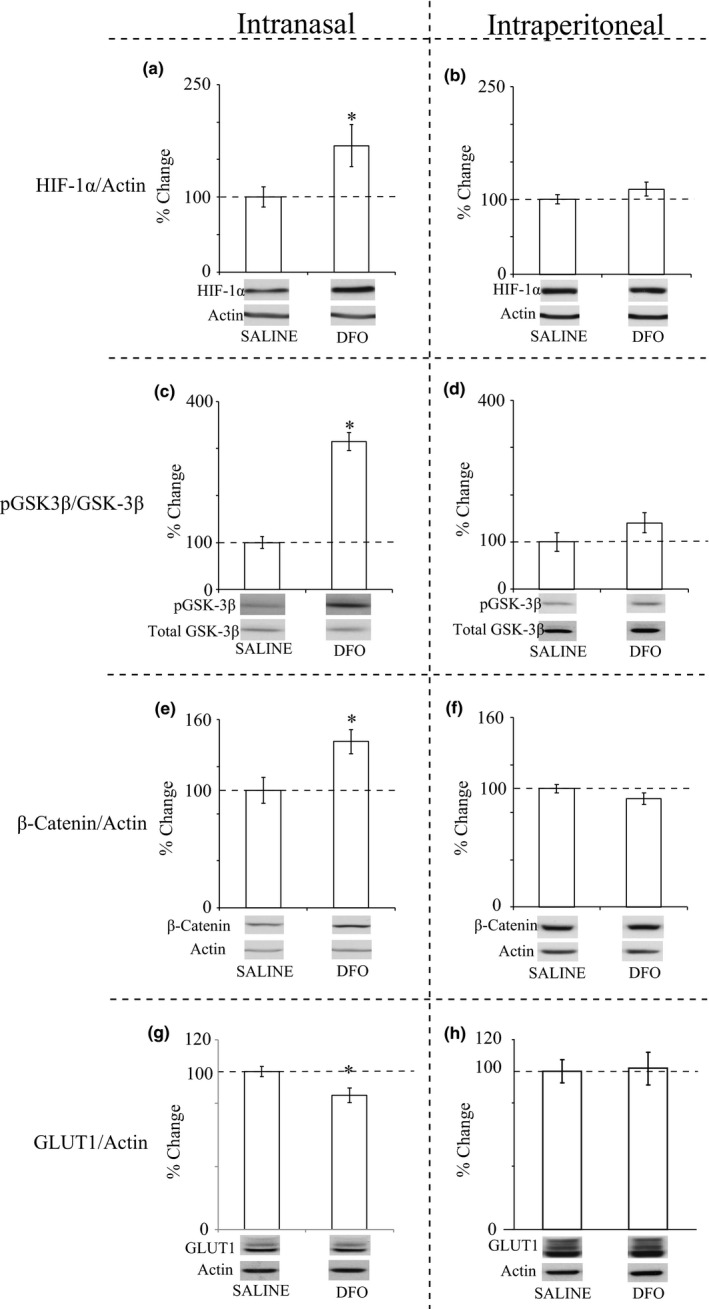
Representative blots and histograms for proteins analyzed by Western blot. C57 mice were treated with DFO either IN (a, c, e, g) or IP (b, d, f, h) for 1 month. (a, b) HIF‐1α; (c, d) pGSK‐3β/GSK‐3β; (e, f) β‐catenin; (g, h) GLUT‐1. Blots are representative of each treatment group, while histograms are percentage of change from vehicle‐treated mice. Error bars are *SEM* converted from the raw data to percentages and are shown only to help visualize differences. Statistically significant differences between groups were measured by *t* test (*p < .05), which was performed on the raw data as optical density rather than percentage changes. IN DFO led to significant increases in HIF‐1α, pGSK‐3β/GSK‐3β, and β‐catenin and decreased GLUT‐1 (*p* < .05). No changes were significant in the IP DFO‐treated mice

Analyses of homogenized hippocampus with Western blotting showed that mice treated with IN DFO had increased HIF‐1α (168%; Figure 2a), an increased ratio of pGSK‐3β to total GSK‐3β (314%; Figure 2c), increased β‐catenin (141%; Figure 2e), and decreased GLUT‐1 (85%; Figure 2g) compared with IN saline controls. All of these changes were significant at *p* < .05. Although there were increases in HIF‐1α and pGSK‐3β/GSK‐3β in mice treated with IP DFO compared with IP controls, these changes were not statistically significant (Figure 2b,d,f,h).

### 3.5 General health

There was no difference in weight between IN treatment groups, whose average weights (±*SE*) for DFO and saline groups were 24.7 ± 0.4 and 25.1 ± 0.6 g, respectively. A *t* test showed IP DFO treatment (23.7 ± 0.3 g) resulted in significant weight loss compared with the IP saline‐treated (25.1 ± 0.5 g) mice at time of sacrifice (*p* < .05). There was no mortality, signs of nasal irritation, or physical defects observed in any group.

## 4 DISCUSSION

In this study, we found that IN DFO improved working memory from baseline, increased levels of HIF‐1α, and inhibited GSK‐3β activity through phosphorylation in healthy C57 mice, while IP DFO delivered at the same dose did not result in these significant behavioral and biochemical changes. Collectively, these findings demonstrate that DFO can improve memory in the absence of a diseased state, a result that promotes the theory that DFO may operate through both disease‐specific and non‐disease‐specific mechanisms. Our results also suggest that DFO may hold promise as a memory enhancer or pretreatment, in addition to treatment, for neurodegenerative disease. Once again, we have correlated treatment with DFO and modulation of the HIF‐1α and GSK‐3β pathways, which have been postulated to play an integral role in DFO's effects in animal models of brain injury and neurodegenerative disease. Furthermore, our results demonstrate the greater efficacy of IN delivery of DFO compared with IP, supporting IN administration as a targeted route for drug administration to the brain.

Our current results support previous findings regarding the use of IN DFO in improving learning and memory in multiple rodent Alzheimer's models with corresponding biochemical increases in HIF‐1α and GSK‐3β (Fine et al., 2012, 2015; Hanson et al., 2012). DFO's ability to improve memory from baseline in healthy mice may yield additional insight into its mechanism given that, by definition, memory improvement in healthy mice can only occur through non‐disease‐specific changes (due to the absence of a diseased state). This finding suggests that DFO's memory‐improving effects in previous Alzheimer's studies could be, in part, a result of non‐disease‐specific modification. Moreover, we postulate that the mechanism of DFO's observed impact in several distinct models of neurologic injury and disease could be via one, or a combination of, non‐disease‐specific pathways such as HIF‐1α, GSK‐3β, or other downstream pathways of iron chelation. This hypothesis is justified by the frequent observation of these non‐model‐specific biochemical changes in the absence of disease‐specific changes in most models (Fine et al., 2015; Sorond et al., 2015; Zhang et al., 2013; Zhao & Rempe, 2011) in addition to our current results, which establish that DFO can enact beneficial change from baseline without disease‐related biochemical modulation.

The pathways contributing to DFO's beneficial effects likely include direct iron chelation, activation of HIF‐1α, and inactivation of GSK‐3β via phosphorylation. The rationale for DFO's effects on HIF‐1α as a driver for DFO's neuroprotective effects in brain injury and neurodegenerative disease have been previously enumerated (Fine et al., 2012; Zhang, Yan, Chang, ShiDu Yan, & Shi, 2011). On the other hand, there is a precedent for DFO to exhibit neuroprotective effects independent of HIF‐1α activation (Siddiq et al., 2009; Zhao & Rempe, 2011), and other pathways likely contribute to DFO's broad impact. For instance, treatment with DFO has been shown to result in the phosphorylation of Akt (PKB), a potential route through which DFO might upregulate glycogen synthesis and glucose uptake, and to inactivate GSK‐3β via downstream phosphorylation (Lui et al., 2015; Nicholson & Anderson, 2002). Glucose uptake and increased insulin signaling have been shown by DFO treatment in cancer and rat liver cells (Dongiovanni et al., 2008). An increase in glycogen synthesis and/or glucose uptake plays a role in the improvement in memory and is indeed the main proposed mechanism by which intranasally administered insulin improves memory in clinical trials in both normal healthy adults and Alzheimer's patients (Benedict & Grillo, 2018). We also postulate that GSK‐3β inactivation could play a role in many models in which DFO has had an effect. GSK‐3β has been shown to be a key player in learning and memory (Hooper et al., 2007). The inhibition of GSK‐3β via phosphorylation has been correlated with neuroprotection in brain injury (Leeds et al., 2014), and GSK‐3β is implicated as a factor in Alzheimer's and other neurodegenerative diseases (Grigor'yan, 2014). Thus, the involvement of HIF‐1α and GSK‐3β is strongly suspected in the memory improvement observed in this study and, we postulate, in the beneficial impacts of DFO across neurologic injury and disease models. Alternatively, DFO has also shown disease‐specific benefits in several models. Decreases in amyloid and tau were associated with DFO treatment in several Alzheimer's studies (Fine et al., 2012, 2015; Guo et al., 2013, 2012) and increased preservation of dopaminergic striatal neurons has been demonstrated in Parkinson's models (Febbraro et al., 2013; Fine et al., 2014; Kaur et al., 2003), suggesting an additional layer of complexity to DFO's mechanism.

An additional result of this study was that IP DFO administered at the same dose as the IN DFO did not demonstrate significant behavioral or biochemical effects of the latter compared with controls, suggesting that IN administration more effectively targets DFO to the brain. IN delivery is thought to involve the well‐characterized nose‐to‐brain pathway (Crowe et al., 2018; Dhuria, Hanson, & Frey, 2010; Lochhead, Wolak, Pizzo, & Thorne, 2015) and may be more effective because it bypasses the blood–brain barrier. DFO delivered via the IP route must travel through the bloodstream (in which it has a short half‐life) and cross the blood–brain barrier, ultimately reaching the brain in substantially lower concentrations. One study found that intranasal administration of DFO significantly increased targeting to various regions of the CNS by ~10 times compared to intravenous administration (Hanson et al., 2009). Although the significant changes observed with the IN‐dosed mice in behavior and biochemistry were not seen in the IP DFO mice, there was a nonsignificant trend toward an increase in the ratio of pGSK‐3β to GSK‐3β and, to a lesser extent, HIF‐1α, in the hippocampus of IP DFO mice. Studies suggest that IP DFO may improve memory in mice with either age‐related or iron‐induced memory impairment (de Lima et al., 2008, 2007). However, the doses in these experiments were 3 times greater than those of the current study, potentially explaining the discrepancy. One additional consideration is that administering IP DFO at such high doses may have adverse effects, since DFO acts as a potent iron chelator in the bloodstream. Indeed, in another study of iron overload, IP DFO led to significantly more deaths in healthy mice than in mice with an iron overload (Porter et al., 1991), suggesting that IP DFO as a brain‐related treatment may be unsafe for mice, or ultimately, patients, without systemic iron overload. We observed significant weight loss in the IP DFO group, potentially related to side effects of systemic exposure. This is also clinically relevant in the HI‐DEF clinical trial, in which DFO is being administered as a potential treatment for intracerebral hemorrhage. This trial was discontinued due to the high DFO dose and has been restarted with a lower DFO dose (http://clinicaltrials.gov). As a direct delivery route to the brain, we speculate that IN DFO could be delivered with fewer systemic side effects than IP DFO because of the lower therapeutic dose required.

While we observed that mice given IN DFO significantly improved RAWM performance, we did not record significant differences in their MWM performance. The MWM is designed to detect reference memory, or the ability to learn a task, whereas the RAWM is more difficult and measures working memory, which requires the ability to manipulate the memories at hand (Arendash et al., 2001). In one sense, we would expect the RAWM to be more sensitive to behavioral differences. However, DFO may selectively improve working memory over reference memory. It is also unclear whether the improvements in working memory for IN DFO‐treated mice were the result of chronic or acute dosing, as the mice experienced both acute dosing 30 min before behavioral tests and long‐term dosing Monday through Friday for 4 weeks. This limitation of the study design may be responsible for the lack of significant MWM results as well, as MWM tests were performed starting on the first day of drug dosing for 5 subsequent days (Monday through Friday), whereas RAWM started the next week after 5 days of dosing and continued for 12 successive days after a greater, chronic exposure to IN DFO. Another caveat to this study's results is that, while HIF‐1α levels increased, GLUT‐1, a downstream target, decreased—contrary to expectations and for unclear reasons. The decreases in GSK‐3β were, however, accompanied by increases in its downstream target, β‐catenin, as expected.

Overall, IN DFO has shown promise as a potential treatment for several forms of neurologic injury and disease, including Parkinson's and Alzheimer's diseases. In this study, we found that IN DFO improved memory from baseline, increased levels of HIF‐1α, and inhibited GSK‐3β activity in healthy C57 mice. These results demonstrate that DFO may operate in the absence of a diseased state and through both non‐disease‐related as well as disease‐specific mechanisms, and corroborate several probable pathways through which DFO may operate to enact beneficial change in neurodegenerative disease. Several potential contributory pathways were beyond the scope of this study—for instance, the direct impacts of the chelation of free iron could also be a common underlying pathway through which DFO may act in these models, as supported by its impacts on Fenton chemistry and oxidative stress, with consequent implications in neurodegenerative disease (Ben‐Shachar, Eshel, Finberg, & Youdim, 1991; Fine et al., 2014). DFO's ability to improve memory from baseline may also have implications as a future treatment not only to improve memory in neurodegenerative disease but also to enhance memory in general or as a pretreatment. Consistent with previous studies, our study showed no adverse effects of IN DFO treatment, as indicated by the lack of deaths or weight loss in the IN DFO groups (Febbraro et al., 2013; Fine et al., 2012, 2014, 2017; Hanson et al., 2012). Future studies will aim to further elucidate this treatment's mechanism, validate its safety, and more broadly look into the common pathways underlying neurodegenerative disease.

## CONFLICT OF INTEREST

WHF and LRH are inventors on a patent owned by HealthPartners Institute related to intranasal deferoxamine. All other authors have no competing interests.

## AUTHOR CONTRIBUTIONS

JMF, WHF, and LRH designed the experiments. AMB, JMC, and ALS performed biochemical tests and analyses. JMF, JVT, JK, and KAF did behavior tests and analyses. All authors helped to write the manuscript.

## Data Availability

The datasets generated during and/or analyzed for the current study are available from the corresponding author upon request.

## References

[brb31536-bib-0001] Arendash, G. W. , King, D. L. , Gordon, M. N. , Morgan, D. , Hatcher, J. M. , Hope, C. E. , & Diamond, D. M. (2001). Progressive, age‐related behavioral impairments in transgenic mice carrying both mutant amyloid precursor protein and presenilin‐1 transgenes. Brain Research, 891(1–2), 42–53. 10.1016/S0006-8993(00)03186-3 11164808

[brb31536-bib-0002] Benedict, C. , & Grillo, C. A. (2018). Insulin resistance as a therapeutic target in the treatment of Alzheimer's disease: A state‐of‐the‐art review. Frontiers in Neuroscience, 12, 215 10.3389/fnins.2018.00215 29743868 PMC5932355

[brb31536-bib-0003] Ben‐Shachar, D. , Eshel, G. , Finberg, J. P. , & Youdim, M. B. (1991). The iron chelator desferrioxamine (Desferal) retards 6‐hydroxydopamine‐induced degeneration of nigrostriatal dopamine neurons. Journal of Neurochemistry, 56(4), 1441–1444. 10.1111/j.1471-4159.1991.tb11444.x 1900527

[brb31536-bib-0004] Crapper McLachlan, D. R. , Dalton, A. J. , Kruck, T. P. , Bell, M. Y. , Smith, W. L. , Kalow, W. , & Andrews, D. F. (1991). Intramuscular desferrioxamine in patients with Alzheimer's disease. Lancet, 337(8753), 1304–1308. 1674295 10.1016/0140-6736(91)92978-b

[brb31536-bib-0005] Crowe, T. P. , Greenlee, M. H. W. , Kanthasamy, A. G. , & Hsu, W. H. (2018). Mechanism of intranasal drug delivery directly to the brain. Life Sciences, 195, 44–52. 10.1016/j.lfs.2017.12.025 29277310

[brb31536-bib-0006] de Lima, M. N. M. , Dias, C. P. , Torres, J. P. , Dornelles, A. , Garcia, V. A. , Scalco, F. S. , … Schröder, N. (2008). Reversion of age‐related recognition memory impairment by iron chelation in rats. Neurobiology of Aging, 29(7), 1052–1059. 10.1016/j.neurobiolaging.2007.02.006 17346856

[brb31536-bib-0007] de Lima, M. N. M. , Presti‐Torres, J. , Caldana, F. , Grazziotin, M. M. , Scalco, F. S. , Guimarães, M. R. , … Schröder, N. (2007). Desferoxamine reverses neonatal iron‐induced recognition memory impairment in rats. European Journal of Pharmacology, 570(1–3), 111–114. 10.1016/j.ejphar.2007.06.002 17617402

[brb31536-bib-0008] Dhuria, S. V. , Hanson, L. R. , & Frey, W. H. 2nd (2010). Intranasal delivery to the central nervous system: Mechanisms and experimental considerations. Journal of Pharmaceutical Sciences, 99(4), 1654–1673. 10.1002/jps.21924 19877171

[brb31536-bib-0009] Dongiovanni, P. , Valenti, L. , Ludovica Fracanzani, A. , Gatti, S. , Cairo, G. , & Fargion, S. (2008). Iron depletion by deferoxamine up‐regulates glucose uptake and insulin signaling in hepatoma cells and in rat liver. American Journal of Pathology, 172(3), 738–747. 10.2353/ajpath.2008.070097 18245813 PMC2258266

[brb31536-bib-0010] Febbraro, F. , Andersen, K. J. , Sanchez‐Guajardo, V. , Tentillier, N. , & Romero‐Ramos, M. (2013). Chronic intranasal deferoxamine ameliorates motor defects and pathology in the alpha‐synuclein rAAV Parkinson's model. Experimental Neurology, 247C, 45–58. 10.1016/j.expneurol.2013.03.017 23531432

[brb31536-bib-0011] Fine, J. M. , Baillargeon, A. M. , Renner, D. B. , Hoerster, N. S. , Tokarev, J. , Colton, S. , … Hanson, L. R. (2012). Intranasal deferoxamine improves performance in radial arm water maze, stabilizes HIF‐1alpha, and phosphorylates GSK3beta in P301L tau transgenic mice. Experimental Brain Research, 219(3), 381–390. 10.1007/s00221-012-3101-0 22547371

[brb31536-bib-0012] Fine, J. M. , Forsberg, A. C. , Renner, D. B. , Faltesek, K. A. , Mohan, K. G. , Wong, J. C. , … Hanson, L. R. (2014). Intranasally‐administered deferoxamine mitigates toxicity of 6‐OHDA in a rat model of Parkinsons disease. Brain Research, 1574, 96–104. 10.1016/j.brainres.2014.05.048 24928620

[brb31536-bib-0013] Fine, J. M. , Forsberg, A. C. , Stroebel, B. M. , Faltesek, K. A. , Verden, D. R. , Hamel, K. A. , … Hanson, L. R. (2017). Intranasal deferoxamine affects memory loss, oxidation, and the insulin pathway in the streptozotocin rat model of Alzheimer's disease. Journal of the Neurological Sciences, 380, 164–171. 10.1016/j.jns.2017.07.028 28870559

[brb31536-bib-0014] Fine, J. M. , Renner, D. B. , Forsberg, A. C. , Cameron, R. A. , Galick, B. T. , Le, C. , … Hanson, L. R. (2015). Intranasal deferoxamine engages multiple pathways to decrease memory loss in the APP/PS1 model of amyloid accumulation. Neuroscience Letters, 584, 362–367. 10.1016/j.neulet.2014.11.013 25445365

[brb31536-bib-0015] Freiherr, J. , Hallschmid, M. , Frey, W. H. 2nd , Brünner, Y. F. , Chapman, C. D. , Hölscher, C. , … Benedict, C. (2013). Intranasal insulin as a treatment for Alzheimer's disease: A review of basic research and clinical evidence. CNS Drugs, 27(7), 505–514. 10.1007/s40263-013-0076-8 23719722 PMC3709085

[brb31536-bib-0016] Freret, T. , Valable, S. , Chazalviel, L. , Saulnier, R. , Mackenzie, E. T. , Petit, E. , … Schumann‐Bard, P. (2006). Delayed administration of deferoxamine reduces brain damage and promotes functional recovery after transient focal cerebral ischemia in the rat. European Journal of Neuroscience, 23(7), 1757–1765. 10.1111/j.1460-9568.2006.04699.x 16623832

[brb31536-bib-0017] Grigor'yan, G. A. (2014). The role of glycogen synthase kinase 3 in the mechanisms of learning and memory. Neuroscience and Behavioral Physiology, 44(9), 1051–1058. 10.1007/s11055-014-0023-2

[brb31536-bib-0018] Guo, C. , Wang, P. , Zhong, M. L. , Wang, T. , Huang, X. S. , Li, J. Y. , & Wang, Z. Y. (2013). Deferoxamine inhibits iron induced hippocampal tau phosphorylation in the Alzheimer transgenic mouse brain. Neurochemistry International, 62(2), 165–172. 10.1016/j.neuint.2012.12.005 23262393

[brb31536-bib-0019] Guo, C. , Wang, T. , Zheng, W. , Shan, Z. Y. , Teng, W. P. , & Wang, Z. Y. (2012). Intranasal deferoxamine reverses iron‐induced memory deficits and inhibits amyloidogenic APP processing in a transgenic mouse model of Alzheimer's disease. Neurobiology of Aging, 34(2), 562–575. 10.1016/j.neurobiolaging.2012.05.009 22717236

[brb31536-bib-0020] Hanson, L. R. , Fine, J. M. , Renner, D. B. , Svitak, A. L. , Burns, R. B. , Nguyen, T. M. , … Frey, W. H. (2012). Intranasal delivery of deferoxamine reduces spatial memory loss in APP/PS1 mice. Drug Delivery and Translational Research, 2(3), 160–168. 10.1007/s13346-011-0050-2 25786865

[brb31536-bib-0021] Hanson, L. R. , Roeytenberg, A. , Martinez, P. M. , Coppes, V. G. , Sweet, D. C. , Rao, R. J. , … Panter, S. S. (2009). Intranasal deferoxamine provides increased brain exposure and significant protection in rat ischemic stroke. Journal of Pharmacology and Experimental Therapeutics, 330(3), 679–686. 10.1124/jpet.108.149807 19509317 PMC2729791

[brb31536-bib-0022] Hernandez, F. , Borrell, J. , Guaza, C. , Avila, J. , & Lucas, J. J. (2002). Spatial learning deficit in transgenic mice that conditionally over‐express GSK‐3beta in the brain but do not form tau filaments. Journal of Neurochemistry, 83(6), 1529–1533. 12472906 10.1046/j.1471-4159.2002.01269.x

[brb31536-bib-0023] Hishikawa, T. , Ono, S. , Ogawa, T. , Tokunaga, K. , Sugiu, K. , & Date, I. (2008). Effects of deferoxamine‐activated hypoxia‐inducible factor‐1 on the brainstem after subarachnoid hemorrhage in rats. Neurosurgery, 62(1), 232–240; discussion 240–231. 10.1227/01.NEU.0000311082.88766.33 18300912

[brb31536-bib-0024] Hooper, C. , Markevich, V. , Plattner, F. , Killick, R. , Schofield, E. , Engel, T. , … Lovestone, S. (2007). Glycogen synthase kinase‐3 inhibition is integral to long‐term potentiation. European Journal of Neuroscience, 25(1), 81–86. 10.1111/j.1460-9568.2006.05245.x 17241269

[brb31536-bib-0025] Jacob, A. , Potin, S. , Saubaméa, B. , Crete, D. , Scherrmann, J.‐M. , Curis, E. , … Declèves, X. (2015). Hypoxia interferes with aryl hydrocarbon receptor pathway in hCMEC/D3 human cerebral microvascular endothelial cells. Journal of Neurochemistry, 132(4), 373–383. 10.1111/jnc.12972 25327972

[brb31536-bib-0026] Kaur, D. , Yantiri, F. , Rajagopalan, S. , Kumar, J. , Mo, J. Q. , Boonplueang, R. , … Andersen, J. K. (2003). Genetic or pharmacological iron chelation prevents MPTP‐induced neurotoxicity in vivo: A novel therapy for Parkinson's disease. Neuron, 37(6), 899–909. 10.1016/S0896-6273(03)00126-0 12670420

[brb31536-bib-0027] Leeds, P. R. , Yu, F. , Wang, Z. , Chiu, C.‐T. , Zhang, Y. , Leng, Y. , … Chuang, D.‐M. (2014). A new avenue for lithium: Intervention in traumatic brain injury. ACS Chemical Neuroscience, 5(6), 422–433. 10.1021/cn500040g 24697257 PMC4063503

[brb31536-bib-0028] Levin, E. D. , McClernon, F. J. , & Rezvani, A. H. (2006). Nicotinic effects on cognitive function: Behavioral characterization, pharmacological specification, and anatomic localization. Psychopharmacology, 184(3–4), 523–539. 10.1007/s00213-005-0164-7 16220335

[brb31536-bib-0029] Li, L. , Yin, X. , Ma, N. , Lin, F. , Kong, X. , Chi, J. , & Feng, Z. (2014). Desferrioxamine regulates HIF‐1 alpha expression in neonatal rat brain after hypoxia‐ischemia. American Journal of Translational Research, 6(4), 377–383. 25075254 PMC4113499

[brb31536-bib-0030] Lochhead, J. J. , Wolak, D. J. , Pizzo, M. E. , & Thorne, R. G. (2015). Rapid transport within cerebral perivascular spaces underlies widespread tracer distribution in the brain after intranasal administration. Journal of Cerebral Blood Flow and Metabolism, 35(3), 371–381. 10.1038/jcbfm.2014.215 25492117 PMC4348383

[brb31536-bib-0031] Lui, G. Y. , Kovacevic, Z. , Richardson, V. , Merlot, A. M. , Kalinowski, D. S. , & Richardson, D. R. (2015). Targeting cancer by binding iron: Dissecting cellular signaling pathways. Oncotarget, 6(22), 18748–18779. 10.18632/oncotarget.4349 26125440 PMC4662454

[brb31536-bib-0032] Nicholson, K. M. , & Anderson, N. G. (2002). The protein kinase B/Akt signalling pathway in human malignancy. Cellular Signalling, 14(5), 381–395. 10.1016/s0898-6568(01)00271-6 11882383

[brb31536-bib-0033] Obolensky, A. , Berenshtein, E. , Lederman, M. , Bulvik, B. , Alper‐Pinus, R. , Yaul, R. , … Banin, E. (2011). Zinc‐desferrioxamine attenuates retinal degeneration in the rd10 mouse model of retinitis pigmentosa. Free Radical Biology and Medicine, 51(8), 1482–1491. 10.1016/j.freeradbiomed.2011.07.014 21824515

[brb31536-bib-0034] Porter, J. B. , Hoyes, K. P. , Abeysinghe, R. D. , Brooks, P. N. , Huehns, E. R. , & Hider, R. C. (1991). Comparison of the subacute toxicity and efficacy of 3‐hydroxypyridin‐4‐one iron chelators in overloaded and nonoverloaded mice. Blood, 78(10), 2727–2734. 10.1182/blood.V78.10.2727.2727 1824264

[brb31536-bib-0035] Salcedo‐Tello, P. , Ortiz‐Matamoros, A. , & Arias, C. (2011). GSK3 function in the brain during development, neuronal plasticity, and neurodegeneration. International Journal of Alzheimer's Disease, 2011, 189728 10.4061/2011/189728 PMC3109514 21660241

[brb31536-bib-0036] Schubert, D. , Soucek, T. , & Blouw, B. (2009). The induction of HIF‐1 reduces astrocyte activation by amyloid beta peptide. European Journal of Neuroscience, 29(7), 1323–1334. 10.1111/j.1460-9568.2009.06712.x 19519624 PMC2752839

[brb31536-bib-0037] Siddiq, A. , Aminova, L. R. , Troy, C. M. , Suh, K. , Messer, Z. , Semenza, G. L. , & Ratan, R. R. (2009). Selective inhibition of hypoxia‐inducible factor (HIF) prolyl‐hydroxylase 1 mediates neuroprotection against normoxic oxidative death via HIF‐ and CREB‐independent pathways. Journal of Neuroscience, 29(27), 8828–8838. 10.1523/JNEUROSCI.1779-09.2009 19587290 PMC3290095

[brb31536-bib-0038] Sorond, F. A. , Tan, C. O. , LaRose, S. , Monk, A. D. , Fichorova, R. , Ryan, S. , & Lipsitz, L. A. (2015). Deferoxamine, cerebrovascular hemodynamics, and vascular aging: potential role for hypoxia‐inducible transcription factor‐1‐regulated pathways. Stroke, 46(9), 2576–2583. 10.1161/strokeaha.115.009906 26304864 PMC4551113

[brb31536-bib-0039] Thorne, R. G. , Pronk, G. J. , Padmanabhan, V. , & Frey, W. H. 2nd (2004). Delivery of insulin‐like growth factor‐I to the rat brain and spinal cord along olfactory and trigeminal pathways following intranasal administration. Neuroscience, 127(2), 481–496. 10.1016/j.neuroscience.2004.05.029 15262337

[brb31536-bib-0040] van Praag, H. , Kempermann, G. , & Gage, F. H. (2000). Neural consequences of environmental enrichment. Nature Reviews Neuroscience, 1(3), 191–198. 10.1038/35044558 11257907

[brb31536-bib-0041] Wan, S. , Hua, Y. , Keep, R. F. , Hoff, J. T. , & Xi, G. (2006). Deferoxamine reduces CSF free iron levels following intracerebral hemorrhage. Acta Neurochirurgica. Supplementum, 96, 199–202. 10.1007/3-211-30714-1_43 16671454

[brb31536-bib-0042] Yu, Z. Q. , Jia, Y. , & Chen, G. (2014). Possible involvement of cathepsin B/D and caspase‐3 in deferoxamine‐related neuroprotection of early brain injury after subarachnoid haemorrhage in rats. Neuropathology and Applied Neurobiology, 40(3), 270–283. 10.1111/nan.12091 24117543

[brb31536-bib-0043] Zaman, K. , Ryu, H. , Hall, D. , O'Donovan, K. , Lin, K. I. , Miller, M. P. , … Ratan, R. R. (1999). Protection from oxidative stress‐induced apoptosis in cortical neuronal cultures by iron chelators is associated with enhanced DNA binding of hypoxia‐inducible factor‐1 and ATF‐1/CREB and increased expression of glycolytic enzymes, p21(waf1/cip1), and erythropoietin. Journal of Neuroscience, 19(22), 9821–9830. 10559391 10.1523/JNEUROSCI.19-22-09821.1999 PMC6782985

[brb31536-bib-0044] Zecca, L. , Youdim, M. B. , Riederer, P. , Connor, J. R. , & Crichton, R. R. (2004). Iron, brain ageing and neurodegenerative disorders. Nature Reviews Neuroscience, 5(11), 863–873. 10.1038/nrn1537 15496864

[brb31536-bib-0045] Zhang, L. , Hu, R. , Li, M. , Li, F. , Meng, H. , Zhu, G. , … Feng, H. (2013). Deferoxamine attenuates iron‐induced long‐term neurotoxicity in rats with traumatic brain injury. Neurological Sciences, 34(5), 639–645. 10.1007/s10072-012-1090-1 22538758

[brb31536-bib-0046] Zhang, Z. , Yan, J. , Chang, Y. , ShiDu Yan, S. , & Shi, H. (2011). Hypoxia inducible factor‐1 as a target for neurodegenerative diseases. Current Medicinal Chemistry, 18(28), 4335–4343. 21861815 10.2174/092986711797200426 PMC3213300

[brb31536-bib-0047] Zhao, Y. , & Rempe, D. A. (2011). Prophylactic neuroprotection against stroke: Low‐dose, prolonged treatment with deferoxamine or deferasirox establishes prolonged neuroprotection independent of HIF‐1 function. Journal of Cerebral Blood Flow and Metabolism, 31(6), 1412–1423. 10.1038/jcbfm.2010.230 21245873 PMC3130314

[brb31536-bib-0048] Zhou, F. Q. , & Snider, W. D. (2005). Cell biology. GSK‐3beta and microtubule assembly in axons. Science, 308(5719), 211–214. 15825222 10.1126/science.1110301

